# Impacts of landscape patterns on habitat quality in coal resource-exhausted cities: Spatial–temporal dynamics and non-stationary scale effects

**DOI:** 10.1007/s10661-025-13707-1

**Published:** 2025-02-15

**Authors:** Zixuan Li, Ziqi Xu, Yedong Chen, Sihao Gu, Cheng Li

**Affiliations:** 1 Leibniz Institute of Ecological Urban and Regional Development, 01217 Dresden, Germany; 2 School of Architecture and Design, China University of Mining and Technology, 221116 Xuzhou, China; 3 School of Mechanics and Civil Engineering, China University of Mining and Technology, 221116 Xuzhou, China; 4 School of Architecture and Urban Planning, Nanjing University, 210093 Nanjing, China; 5 School of Architecture, Southeast University, 210096 Nanjing, China

**Keywords:** Landscape patterns, Habitat quality, Resource-exhausted cities, Spatial–temporal dynamics, MGTWR model, Jiawang

## Abstract

Coal resource–based cities were once pillars of significant economic and social development, but after resource exhaustion, these cities are at a critical crossroads and need to transition towards sustainable development and ecological urban renewal. The unique landscape patterns of these cities are associated with extensive coal mining, and their dynamic changes are intrinsically linked to habitat quality. However, this relationship has not been fully explored in existing research. Additionally, the spatial–temporal dynamics and non-stationary scale effects of landscape patterns on habitat quality are often overlooked. This study selects Jiawang, a typical coal resource–exhausted city in eastern China, as a case study. We focus on the ecological transition period from 2000 to 2020. Based on land cover data, the study quantitatively describes the spatiotemporal evolution of landscape patterns and habitat quality. A novel multiscale geographically and temporally weighted regression (MGTWR) model is used to analyze and quantify the complex effects of landscape patterns on habitat quality at different spatiotemporal scales. The study further elucidates the dynamic interaction between landscape patterns and habitat quality, emphasizing key non-stationary scale effects. The findings provide insights for strategic ecosystem management and spatial planning, offering a blueprint for the sustainable transformation of coal resource-exhausted cities.

## Introduction

The development of coal resource-based cities is closely related to resource extraction activities, which have made significant contributions to economic and social development (Chen et al., 2019). The exhaustion of resources is a critical turning point in the life cycle of such cities (Mao & He, 2008; Shang et al., 2022). Coal resource–exhausted cities face two fates: transformation and rejuvenation or contraction and decline. Guided by the concept of sustainable development, urban ecological transformation and the development of a green economy are important paths for the regeneration of cities depleted of coal resources (Gu et al., 2022). The landscape patterns of Coal resource-exhausted cities have unique characteristics due to the history of coal mining and its ongoing impact. Due to extensive mining activities, these cities often exhibit problems such as increased landscape fragmentation, reduced landscape connectivity, and enhanced landscape heterogeneity (Z. Wu et al., 2019a, 2019b). Such changes in the landscape can lead to significant alterations in farmland, water bodies, and impervious surfaces. At the same time, the reuse of mining sites and the transformation and regeneration of industries have promoted the renewal of urban land use. These factors create a clear spatiotemporal heterogeneity in the urban landscape pattern (Guan & Yu, 2021; Zhang et al., 2021). Moreover, the habitat quality of such cities is closely related to the landscape pattern. Continuous large-scale coal mining can lead to damage to the farmland around the mines, subsidence forming pits and ponds, landslides, ground fissures, and other geological disasters, thereby affecting the overall habitat quality (Guan & Yu, 2021). It is worth noting that coal mining subsidence areas, often seen as secondary anthropogenic disasters, can, after ecological landscape reconstruction, become secondary habitats with high conservation value, replacing rapidly diminishing natural wetlands (Zhang et al., 2019). It is evident that the habitat quality of these cities exhibits significant heterogeneity in both time and space. Therefore, exploring the impact of landscape patterns of coal resource-exhausted cities on habitat quality is crucial for understanding and assessing the long-term effects of coal mining on the ecological environment. Simultaneously, this helps provide practical suggestions for promoting effective ecosystem management and spatial planning.

Habitat quality refers to the environmental conditions in an area where biological communities can live and reproduce suitably. It encompasses various elements, including the overall integrity of natural ecosystems, the availability of resources, and the influence of humans activities on the ecosystem (Gong et al., 2019; Hatziiordanou et al., 2019). The compromise of habitat quality can result in a decrease in biodiversity, a decline in ecosystem services, and subsequently impact the equilibrium of ecological systems and the well-being of human (Zhao et al., 2022). The assessment of habitat quality can mainly be divided into two aspects. On the one hand, traditional methods primarily use the resources available in habitats or the number and density of target organisms within an area for measurement. This approach is labor-intensive and difficult to conduct over extensive geographic regions. On the other hand, with the development of remote sensing and geographic information technology, numerous models have emerged that use land cover data to calculate regional habitat quality across broad spatial extents, such as the Maximum Entropy Model (MaxEnt) and the integrated assessment and trade-off of ecosystem services based on their societal value (InVEST) (D. Li et al., 2021a, 2021b; Sallustio et al., 2017). The habitat quality of coal resource-exhausted cities is closely related to the distribution of mines and coal-related industries (Li et al., 2023a, 2023b). Using the InVEST model can, based on the specificity of its development, input appropriate parameters and coal mine–related threat sources to accurately calculate the results of habitat quality. Furthermore, this model can quickly calculate the regional habitat quality over a large area and for many years, overcoming the difficulty of verifying historical habitat quality, and can also reflect the dynamic impact of coal resource extraction on habitat quality, providing a reliable guarantee for longitudinal studies.

The concept of landscape patterns pertain to the arrangement, composition, and interconnectedness of diverse landscape components within a region. It serves as a representation of the ecological organization and spatial arrangement of land cover within the area (Tischendorf, 2001; Turner, 1989). To quantify and analyze changes in landscape patterns, scholars typically use landscape metrics to describe the patterns from multiple aspects such as edge, shape, aggregation, and diversity (Tischendorf, 2001). Landscape metrics are divided into three different levels: individual patches, specific types of patches, and the entire landscape. These three levels of indices provide consistent criteria for measuring various aspects of landscape patterns. This hierarchical structure allows us to observe the dynamic changes in landscape patterns, as well as to compare landscape patterns across different geographical spaces and time periods (Fu et al., 2011; Hamstead et al., 2016). This can effectively and accurately describe the dynamic changes in the landscape patterns of resource-exhausted cities. Additionally, this hierarchical structure also allows for the possibility of using statistical methods to quantitatively study the key relationships between landscape patterns and other factors.

For decades, the relationship between landscape patterns and habitat quality has been the focus of research in landscape ecology, ecology, and geography. Scholars have noted that regional landscape patterns have a greater impact on biodiversity than the internal structure of habitats (Hou et al., 2020). Changes in landscape patterns can lead to deterministic changes in habitats, resulting in significant alterations in community composition (Parody et al., 2001). Also, changes in the shape and spatial arrangement of landscape patches are significantly related to landscape fragmentation and connectivity, as well as to changes in the provision of ecosystem services (Guo et al., 2022). In summary, changes in regional landscape patterns directly affect changes in habitat quality, necessitating continuous monitoring and adaptive strategies to ensure ecological balance (Liu et al., 2022).

Traditional linear regression models are widely used to investigate the relationships between explanatory variables and independent variables across various fields, including ordinary least squares (Stewart Fotheringham et al., 1996; Sun et al., 2019; Wang et al., 2019), correlation analysis (Zhang et al., 2020), multiple linear regression (Hou et al., 2020), stepwise regression (Hao et al., 2017), and redundancy analysis (Shen et al., 2021).These methods are based on the premise that statistical data are stable across the entire dataset. However, they do not consider geographical heterogeneity and local differences, which may lead to model biases and limitations in detecting the impact of landscape patterns on habitat quality (Li et al., 2017). In response to the non-stationarity of geographical spatial data, geographically weighted regression (GWR) model has evolved as a prominent approach that has garnered increasing popularity (Brunsdon et al., 1998). The GWR model is a statistical tool that utilizes local regression equations to analyze spatial interactions. By incorporating many variables, it offers a more comprehensive understanding of complicated spatial patterns. Consequently, the GWR model enhances the precision and explanatory capacity of spatial variables (Su et al., 2012). Nevertheless, the conventional GWR model has a deficiency in considering the temporal dimension of the data and encounters challenges related to the varying scales of explanatory variables (Bai et al., 2019; Zhao & Li, 2022). In order to tackle the aforementioned issues, two extensions of GWR model were introduced, namely the geographically and temporally weighted regression (GTWR) model (Fotheringham et al., 2015; Huang et al., 2010) and the multiscale geographically weighted regression (MGWR) model (Fotheringham et al., 2017). On the one hand, the GTWR model incorporates the temporal dimension of variables, enhancing the model’s effectiveness, particularly suitable for time-series analysis (Fotheringham et al., 2015; Huang et al., 2010). Landscape dynamics are an ongoing temporal process where current changes are influenced by past dynamics and, in turn, affect future ecological outcomes. Using GTWR model to analyze related long-term sequence data can reveal patterns of dynamic change, providing predictions and recommendations for future development. On the other hand, the MGWR model aims to determine the ideal bandwidth for each explanatory variable, thereby broadening the scope of explanatory variables (Fotheringham et al., 2017). Combining the spatial dimensions of variables and pursuing the optimal bandwidth for each explanatory variable presents a novel and complex issue (Hu et al., 2022). In landscape ecology, different elements have different scales of effect. The application of the MGWR model allows us to explore the strength of different scales and variables within the same landscape ecosystem, thereby providing a strong reference for studying its spatial effects.

The studies mentioned above show that currently, there is a research gap in the non-stationary scale effects on habitat quality of coal resource-exhausted cities in the spatiotemporal dynamics response of landscape patterns. The concept of scale is an important issue in geography (Johnston, 1969; Tate & Atkinson, 2001). Spatial and temporal phenomena are inherently influenced by scale effects (Gao & Bian, 2016). Firstly, the MGWR model does not consider the continuous impact of temporal fluctuations in landscape patterns on habitat quality. Secondly, the GTWR model uses a single average bandwidth to calculate the range of variables’ effects, ignoring the spatiotemporal scale differences of various landscape metrics, which may exaggerate or underestimate the changes in these spatiotemporal effects. They have not integrated each other’s strengths. Therefore, the multiscale geographically and temporally weighted regression (MGTWR) has been proposed to address this issue (C. Wu et al., 2019a, 2019b). Utilizing the MGTWR model can more accurately examine the impacts of various explanatory variables on habitat quality across multiple temporal and geographic scales, resolving the issue of inconsistent model coefficients when analyzing GTWR and MGWR in two steps (Hu et al., 2022). This allows for a more comprehensive understanding of the complex mechanisms behind the association between landscape patterns and habitat quality.

In this study, we selected Jiawang, a typical coal resource–exhausted city in eastern China, as our study area. The city’s coal mining activities date back to 1882, and the exploitation of coal resources continued until they were finally exhausted in 2000. After the exhaustion of coal resources, the city underwent rapid urbanization and implemented a series of ecological restoration projects. Significant changes have occurred in its landscape pattern and habitat quality. To fill the aforementioned research gap, this study aims to investigate the spatiotemporal instability of the effects of landscape patterns on habitat quality in coal resource-exhausted cities, as well as the scale effects during the impact process. The specific objectives of this study are as follows:

1. To reveal the spatiotemporal characteristics of landscape patterns and habitat quality in the Jiawang district from 2000 to 2020.
2. To discuss the spatiotemporal dynamic response of habitat quality to landscape patterns and the non-stationary scale effects.
3. To provide insights for effective ecosystem management and spatial planning.

## Study area and data sources

### Study area

**Fig. 1:**
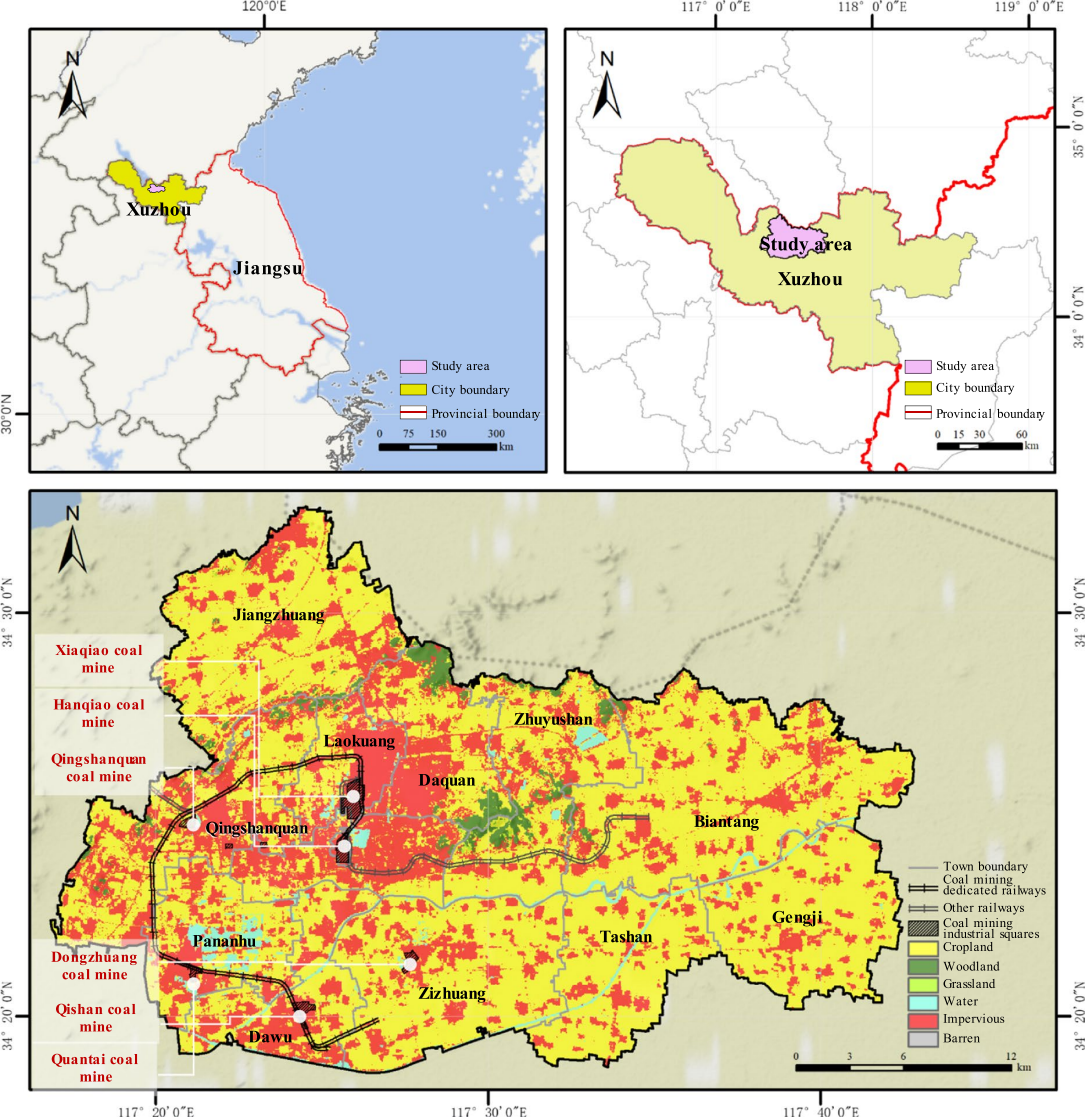
Location of the research area

The Jiawang District (Longitude 117°17′ ~ 117°42′, Latitude 34°17′ ~ 34°32′) is located in the northeast of Xuzhou City, Jiangsu Province, China, covering an area of 612 km^2^ (Fig. 1). The geography of Jiawang exhibits an elevation disparity, with the western region being higher than the eastern region, and the northern region being higher than the southern region. Additionally, there is a gentle slope towards the southeast. Jiawang’s urban development is closely related to coal resource extraction. Since the extraction of coal resources in 1882, Jiawang has developed into a city because of the mines. At its peak, it had 262 coal mines of various sizes. Among them, there are many important state-owned coal mines, such as Xiaqiao, Hanqiao, and Qishan coal mines. However, since 2000, coal reserves have been increasingly depleted. Policy measures in 2001 led to the closure of over 120 small coal kilns, and the major coal mines also gradually exhausted their resources. In 2011, Jiawang was listed by the nation as the only resource-exhausted city in Jiangsu Province, representing coal resource–exhausted cities. Relying on coal resources, Jiawang’s socio-economic prosperity flourished. With the exhaustion of resources, the city’s development lost its momentum. Meanwhile, resource extraction has had a significant impact on the structure and processes of the ecosystem. The contradiction between ecological safety and socio-economic development has become a major obstacle to Jiawang’s sustainable development.

In recent years, under the influence of national and local policies, Jiawang has gradually shed its reliance on resource industries and implemented a large number of ecological restoration projects. This has resulted in good ecological transformation outcomes, making it a national model for resource-based urban transformation. In the transformation process over the past twenty years, Jiawang’s landscape pattern has undergone significant changes, and the habitat quality of coal mining-damaged areas has been improved. It provides a model for studying the spatiotemporal dynamic relationship between landscape patterns and habitat quality in coal resource-exhausted cities. Meanwhile, understanding the complex impact of landscape patterns on habitat quality helps provide practical suggestions for Jiawang’s sustainable transformation.

### Data sources

For this study, we utilized the following data resources:

1. Land cover data of Jiawang: The study employed data from the years 2000, 2005, 2010, 2015, and 2020. This data, with a spatial resolution of 30 m, comes from the CLCD dataset developed by Wuhan University (Yang & Huang, 2021). The overall accuracy of this dataset is 79.31%, and it is interpreted based on the Landsat images. Land cover data of Jiawang were reclassified as cropland (including dry and paddy fields), woodland (including forest and shrubs), grassland, water, impervious (including urban construction land, rural settlements, industrial and mining land) and barren.
2. Vector boundary data of coal mine industrial squares: This data is interpreted based on historical literature and historical imagery from Google.
3. Railway vector data: This includes railways dedicated to coal mines and other railways, and is also interpreted based on historical literature and historical imagery from Google.
4. Administrative division data: This data was directly obtained from the Jiawang Natural Resources and Planning Bureau, including the administrative division information of Jiawang District and its subordinate townships.

## Methodology

**Fig. 2:**
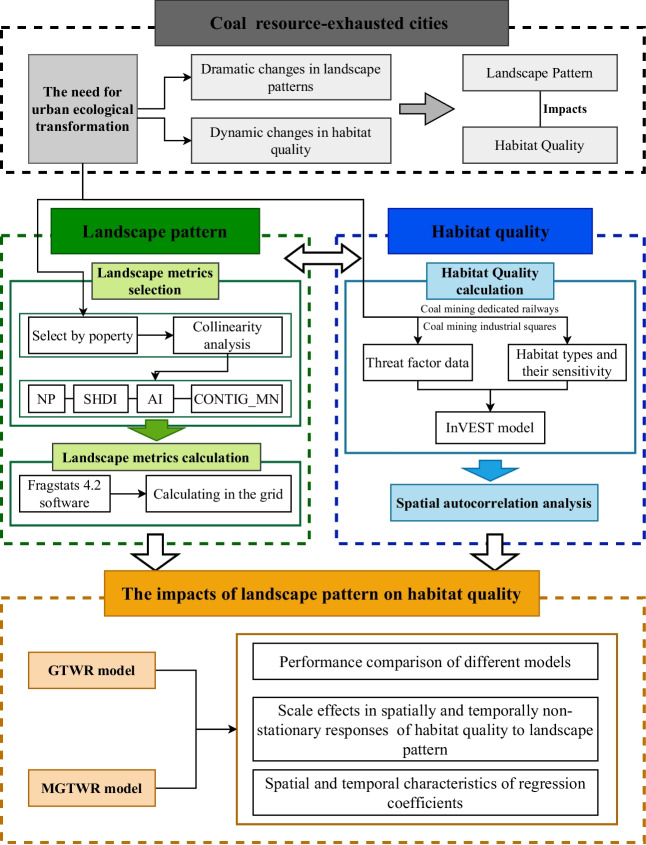
The methodological framework of the study. Abbreviations represent selected landscape metrics: number of patches (NP), mean contiguity index (CONTIG_MN), Shannon’s diversity index (SHDI), and aggregation index (AI)

The methodological framework of the study is illustrated in Fig. 2. Under the practical need for ecological transformation in coal resource–exhausted cities, this study investigated the dynamic impact of landscape patterns on habitat quality and the scale effects from 2000 to 2020. Firstly, we selected appropriate landscape metrics for the study area, and used Fragstats software to calculate and visualize them in a grid; secondly, given the uniqueness of coal resource-exhausted cities, we applied the InVEST model to accurately quantify habitat quality in the study area, and conducted spatial autocorrelation analysis of spatial clustering patterns; Lastly, compared the performance of GTWR and MGTWR models and used the MGTWR model to analyze the spatiotemporal characteristics and scale effects of landscape patterns on habitat quality.

### Selection and calculation of landscape metrics

The selection and combination of landscape metrics play a pivotal role in comprehensively capturing and representing landscape patterns. The criteria for selecting landscape pattern indices in this study are as follows:

1. The combination of landscape metrics should comprehensively reflect the characteristics and structure of landscape patterns.
2. The selection of metrics should meet three key criteria: alignment with current research, acceptance by the scientific community, and representativeness (Hu et al., 2022; Li et al., 2023a, 2023b).
3. Metrics should avoid redundancy (Lei et al., 2021).
4. Metrics should be simple to understand and relevant to the characteristics of the study area (Mei et al., 2019).

To fully reflect Jiawang’s landscape pattern, we chosen four landscape metrics at the landscape level: number of patches (NP), mean contiguity index (CONTIG_MN), Shannon’s diversity index (SHDI), and aggregation index (AI). These metrics were chosen based on Jiawang’s characteristics as a coal mining city and in combination with pertinent existing research. The complexity, connectedness, diversity, and aggregation of the landscape can be reflected in these metrics. Additionally, to reduce multicollinearity and guarantee the robust performance of subsequent regression models, all metrics have a Variance Inflation Factor (VIF) 5 to minimize multicollinearity (Tay, 2017).

To analyze the study area, we applied a grid-based sampling method using ArcGIS 10.5, dividing the landscape into 500 m × 500 m grid cells (Li & Shuai 2023).This process resulted in 2637 sampling points, with each point representing the centroid of a grid cell. Landscape metrics were subsequently computed for each grid cell at the 500-m scale. Notably, these sampling points were generated based on the geometric centroids of the grid cells, ensuring spatial consistency in subsequent analyses.

### Calculation of habitat quality

The InVEST model calculates habitat quality of a region by integrating land type sensitivity and external threat intensity. Its computation requires relatively minimal data and offers a clear and intuitive representation of biodiversity functionality. It has been widely utilized in the fields of geography and ecology (Caro et al., 2020; Wu et al., 2021). This study calculates the habitat quality of the study area using the Habitat Quality module in InVEST 3.11.0. Table 1 lists the threat factor data, while Table 2 displays the habitat types and their sensitivity to threats. Based on existing relevant research and the actual situation of Jiawang as a coal mining city, cropland, impervious surfaces, coal mining industrial squares, coal mine dedicated railways, and other railways were designated as threat sources. Woodland, grassland, water, cropland, and barren land were considered as habitat-providing land types (Bian & Lu, 2013; Li et al., 2023a, 2023b). Due to the closure of all coal mines in the study area in 2016, the coal mining industrial squares are no longer considered as threat sources when calculating Jiawang’s habitat quality in 2020. Habitat quality ${Q}_{xj}$ is calculated by the following formula:

1 $${Q}_{xj}={H}_{j}\left[1-\frac{{D}_{xj}^{Z}}{{D}_{xj}^{Z}+{k}^{z}}\right]$$

where ${Q}_{xj}$: habitat quality of raster x in land cover type $j$; ${H}_{j}$: the habitat suitability of land cover type $j$; ${D}_{xj}$: total threat level of raster x in land cover type $j$; $Z$: a scaling parameter and is 2.5 in this paper; $k$: a half-saturation constant, typically assigned a value of 0.5.

**Table 1:** Threat factor parameter setting

Threat	MAX DIST	Weight	Decay
Cropland	1.5	0.7	exponential
Barren	1	0.4	exponential
Impervious	4	1	exponential
Coal mining industrial squares	6	1	exponential
Coal mine dedicated railways	2	0.8	exponential
Other_railways	1.5	0.6	exponential

**Table 2:** Sensitivity of land-cover types to each threat

Land cover	Habitat	Threat factor					
		Cropland	Barren	Impervious	Coal mining industrial squares	Coal mine dedicated railways	Other railways
Nodata	0	0	0	0	0	0	0
Cropland	0.4	0.3	0.1	0.5	0.6	0.45	0.3
Woodland	0.95	0.45	0.3	0.6	0.7	0.6	0.4
Grassland	0.65	0.4	0.25	0.5	0.6	0.4	0.25
Water	0.8	0.65	0.2	0.7	0.75	0.6	0.4
Impervious	0	0	0	0	0	0	0
Barren	0.2	0.2	0.2	0.1	0.5	0.4	0.2

### Spatial autocorrelation analysis

Spatial autocorrelation analysis is a method used to monitor whether there is a dependency relationship between geographical spatial data values (Liu et al., 2021). It reveals trends and patterns in spatial distribution. The Global Moran’s index reflects the spatial clustering or dispersion of data across the entire region (Fu et al., 2014). The Local Moran’s index, on the other hand, analyzes the spatial correlation between specific locations and their neighboring points, thereby revealing local clustering patterns in spatial data (Anselin, 1995). In this study, to measure and test the overall and local clustering characteristics of habitat quality, we calculated the Global Moran’s I and Local Moran’s I for the habitat quality in the study area based on GeoDA software, using 500-m grid cells as the spatial units, consistent with the grid size selection described earlier. The formulas for their calculation are as follows:

2 $$\begin{array}{c} \, {\mathrm{G}}{\mathrm{l}}{\mathrm{o}}{\mathrm{b}}{\mathrm{a}}{\mathrm{l}} \, {\mathrm{M}}{\mathrm{o}}{\mathrm{r}}{\mathrm{a}}{\mathrm{n}}{\prime} \, s I=\frac{n\sum_{i=1}^{n} \sum_{j=1}^{n} {w}_{ij}\left({x}_{i}-\overline{x }\right)\left({x}_{j}-\overline{x }\right)}{\sum_{i=1}^{n} \sum_{j=1}^{n} {w}_{ij}\sum_{i=1}^{n} {\left({x}_{i}-\overline{x }\right)}^{2}}\end{array}$$

3 $$\begin{array}{c}{\mathrm{ Local Moran }}^{\mathrm{'}}s I=\frac{{x}_{i}-\overline{x}}{{\sigma }^{2}}\sum_{j=1,j\ne i}^{n} \left[{w}_{ij}\left({x}_{j}-\overline{x }\right)\right]\end{array}$$

where $n$ represents the number of spatial units; ${w}_{ij}$ is the weight between locations $i$ and $j$; ${y}_{i}$ and ${y}_{j}$ represent the selected attribute values at locations $i$ and $j$, respectively; $\overline{y }$ is the average value of all observations. Moran’s I ranges from − 1 to 1. A positive index indicates spatial correlation, while a negative index indicates spatial heterogeneity.

### Regression models

#### Geographically and temporally weighted regression

Before introducing the MGTWR model, it is necessary to introduce the theoretical method of the GTWR model, which helps to deepen the understanding of the spatiotemporal modeling process. The GTWR model, building on the consideration of local spatial heterogeneity in the GWR model, introduces the time dimension (Huang et al., 2010). It employs neighboring sample points in both time and space domains to calibrate regression coefficients for local observation points, thus enabling the analysis of spatiotemporal non-stationarity. Changes in landscape patterns and habitat quality are represented by panel data with multiple time series, and changes in landscape patterns do not immediately result in changes in habitat quality, their impact may have a certain lag effect (Du Toit et al., 2016). Therefore, in this study, the GTWR model was utilized to explore the spatiotemporal dynamic impacts of landscape patterns on habitat quality, laying the groundwork for subsequent comparison with regression results from the MGTWR model. The specific formula is as follows:

4 $${\mathrm{Y}}_{i}={\beta }_{0}\left({u}_{i},{v}_{i},{t}_{i}\right)+\sum_{k=1}^{m} {\beta }_{k}\left({u}_{i},{v}_{i},{t}_{i}\right){x}_{ik}+{\varepsilon }_{i}$$

where (${u}_{i}$,${v}_{i}$,${t}_{i}$) is the sample point $i$ with spatial coordinates and time stamps; $m$ is the number of samples; ${\varepsilon }_{i}$ is the random error term, and ${\beta }_{k}$ (${u}_{i}$,${v}_{i}$,${t}_{i}$) are the estimated local regression coefficients.

#### Multiscale geographically and temporally weighted regression

The Multiscale Geographically and Temporally Weighted Regression (MGTWR) model is an extension of the GTWR model. An apparent limitation of the GTWR model is that it employs an average spatiotemporal bandwidth as the influence range for each explanatory variable. However, the scale effects of each landscape metric on habitat quality cannot be entirely consistent, and using the same spatiotemporal bandwidth may overestimate or underestimate the scale of the variable’s effect. The MGTWR model allows each individual variable to have different spatiotemporal stationarity and permits variables to have specific bandwidths, addressing the issue in the GTWR model where all variables are constrained to the same optimal bandwidth (C. Wu et al., 2019a, 2019b). Therefore, this study used the MGTWR model to further explore the scale effects of landscape patterns on the spatiotemporal impact on habitat quality. The specific formula is as follows:

5 $$\begin{aligned}{y}_{i}&={\beta }_{\mathrm{bst}(0)}\left({u}_{i},{v}_{i},{t}_{i}\right)+\sum_{k=1}^{K} {\beta }_{\mathrm{bst}(k)}\left({u}_{i},{v}_{i},{t}_{i}\right){x}_{ik}+{\varepsilon }_{i}\\&=\sum_{k=0}^{K} {f}_{k}+{\varepsilon }_{i}\end{aligned}$$

where $k$ represents the number of explanatory variables; $\mathrm{bst}(k)$ is the specific spatiotemporal bandwidth of the explanatory variable $k$; ${\beta }_{\mathrm{bst}(k)}$ is the regression coefficient of the explanatory variable $k$ for the specific spatio-temporal bandwidth; ${f}_{k}$=${\beta }_{\mathrm{bst}(\mathrm{k})}\left({u}_{i},{v}_{i},{t}_{i}\right)$ is additive term of the explanatory variable $k$.

The MGTWR model is a generalized additive model. In order to find the optimal spatial and temporal bandwidth, the GTWR model is used to initialize the additive term vector first. The error term for this process is as follows: $\widehat{\varepsilon }=y-\sum_{k=0}^{K} {f}_{k}$. Then, the backward fitting algorithm is then used to iteratively update to find the optimal bandwidth for each variable until the update stops when the convergence criterion is reached. The convergence criterion is the classical SOC_RSS_ using ≤ 10^−5^ as the termination criterion (C. Wu et al., 2019a, 2019b), which is given in the following equation:

6 $${\mathrm{SOC}}_{\mathrm{RSS}}^{k+1}=\left|\frac{{\mathrm{RSS}}^{k+1}-{\mathrm{RSS}}^{k}}{{\mathrm{RSS}}^{k+1}}\right|$$

where ${\mathrm{RSS}}^{k+1}$ represents the sum of squares of the residuals of round $k$+1; ${\mathrm{RSS}}^{k}$ represents the sum of squares of the residuals of round $k$.

## Results

### Analysis of the spatial and temporal changes of landscape patterns

#### Spatial characteristics of land cover

**Fig. 3:**
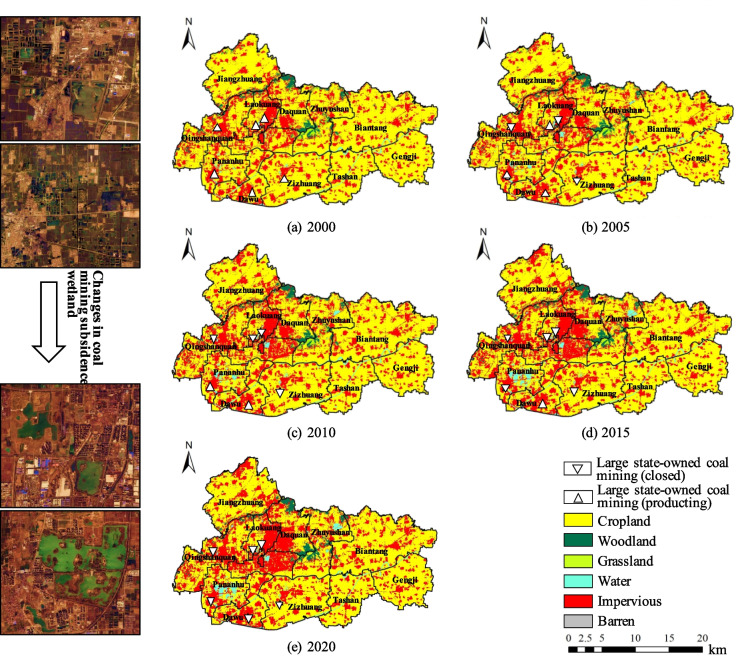
Changes in the land cover pattern

Over the course of two decades following the exhaustion of coal resources, Jiawang had significant transformations in land cover (Fig. 3). In 2000, Jiawang was still a city largely dependent on coal mining for its development. During this period, the main type of land cover in Jiawang was cropland land, which was widely distributed. Large areas of impervious surface were scattered in their overall spatial characteristics, mainly concentrated in the central, western, and southwestern parts of the study area. The central urban area and main towns had a close spatial relationship with the large state-owned coal mines. The central and northern regions of Jiawang were mainly woodland and grassland, representing the mountainous and hilly terrain of the area. Water bodies were primarily composed of rivers, traversing the study area from the southwest to the east, all part of the main routes and tributaries of the Beijing-Hangzhou Grand Canal. There were small and fragmented bodies of water distributed around large impervious surfaces, all of which were coal mining subsidence wetlands caused by mining activities. With the exhaustion of resources, Jiawang began its urban transformation and development. During these 20 years, the urbanization pace in Jiawang accelerated. The central urban area started to expand eastward and southward, with a new urban center emerging in the southern part of the city. The impervious surfaces exhibited a multicentric overall spatial structure. From the perspective of ecological land use, the spatial characteristics of woodland and grassland did not change much, with some reversion of grassland to farmland in the central mountainous and hilly areas. Notably, the spatial characteristics of water bodies such as lakes and wetlands changed significantly. The fragmented water bodies surrounding large impervious surfaces evolved towards areal aggregation, forming extensive lakes and wetlands. This is a positive manifestation of the ecological transformation in the study area.

#### Transfer analysis of land cover

**Fig. 4:**
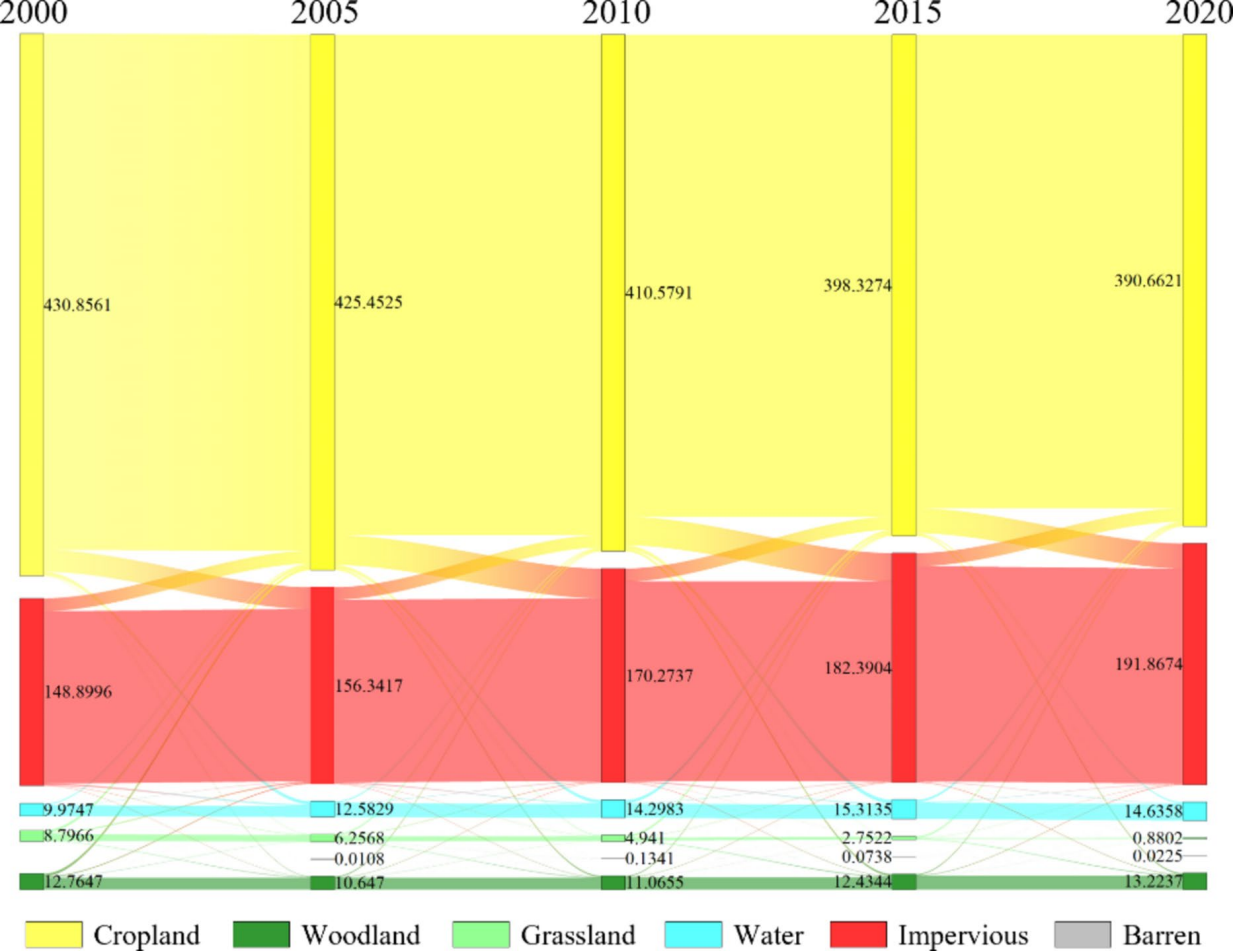
Transfer process of various land cover types (unit:km^2^)

The transitions between various land cover types from 2000 to 2020 are shown in Fig. 4. Following resource depletion, Jiawang entered a phase of urban transformation and development, marked by a notable increase in the area of impervious surfaces. This was accompanied by the encroachment of large areas of cropland. By 2020, the area of impervious surfaces was 1.29 times that of 2000, an increase of 42.96 km^2^. Similarly, during this period, a total of 40.194 km^2^ of cropland was lost, averaging a decrease of 2 km^2^ per year. Of this, a total of 42.55 km^2^ of arable land was transformed into impervious surfaces. The substantial increase in impervious surfaces poses potential risks to habitat quality in the study area and affects the overall ecological security of the region. Moreover, over these 20 years, Jiawang has implemented a series of ecological restoration projects, resulting in an increase in woodland and water areas. Although the woodland area decreased in 2005, overall, by 2020, Jiawang’s woodland area increased by 0.46 km^2^ compared to 2000. The expansion of water areas is a significant reflection of Jiawang’s ecological transformation, with an overall increase of 4.66 km^2^. The increase in these ecological lands has a positive effect on the habitat quality of the study area.

#### Analysis of the spatial and temporal changes of landscape metrics

**Fig. 5:**
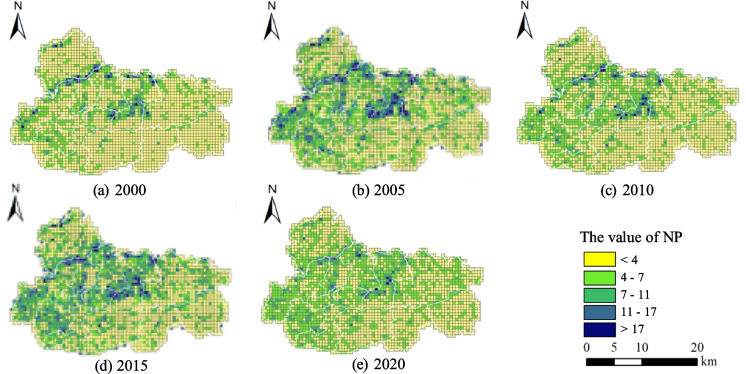
Changes in number of patches (NP) pattern

As shown in Fig. 5, Jiawang’s number of patches (NP) value demonstrates a spatial distribution pattern characterized by lower values overall, with a higher concentration in the western region and a lower concentration in the eastern region. The center and northern mountainous regions exhibit a notable concentration of high NP values surpassing 17. Conversely, the eastern parts, characterized by continuous cropland, predominantly display low NP values, falling below 4. This distribution pattern is primarily influenced by the prevailing landscape type. From a temporal perspective, there has been a decline in the quantity of grids in Jiawang that possess low NP values (less than 4), however the number of grids exhibiting relatively high NP values (ranging from 4 to 17) has demonstrated a consistent upward tendency over the years. In specific areas, there have been significant changes in NP values. The NP values in the northwest region had a general trend of low values in 2000. However, by 2020, there was an observable clustering phenomenon of grids displaying higher NP values.

**Fig. 6:**
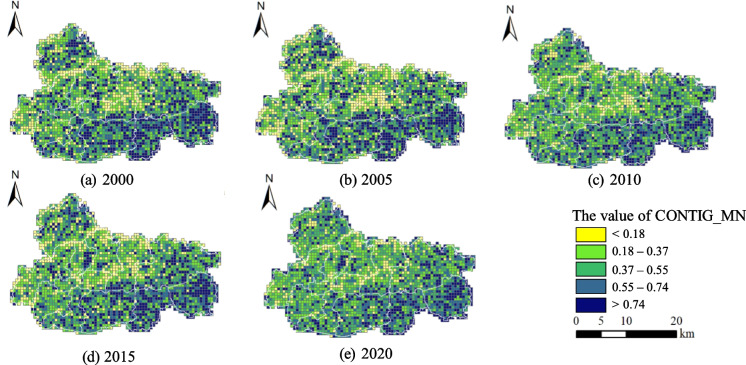
Changes in mean contiguity index (CONTIG_MN) pattern

The mean contiguity index (CONTIG_MN) value of Jiawang tends to be high, with a majority of grids having values more than 0.37 (Fig. 6). Locations with high CONTIG_MN values, exceeding 0.74, are predominantly situated in the southern agricultural and central river regions. Conversely, low values, below 0.37, are observed in locations where urban and agriculture zones intersect. The potential cause for the occurrence of grids with low values could be attributed to the presence of fragmented coal mine subsidence wetlands. As the city has undergone transformative development, there has been a steady improvement in areas with inadequate connectivity. In the core part of Jiawang, there has been a noticeable decline in the clustering phenomena of low-value grids, namely those with a CONTIG_MN value below 0.37. Additionally, the presence of randomly distributed low-value grids towards the edges has gradually diminished.

**Fig. 7:**
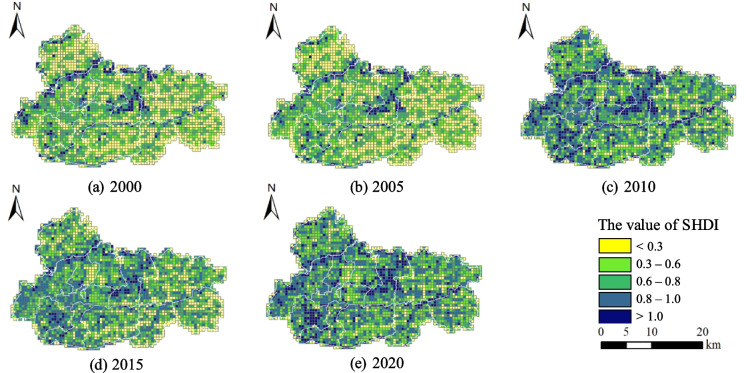
Changes in Shannon’s diversity index (SHDI) pattern

As shown in Fig. 7, there is a consistent increase trend observed in Jiawang’s Shannon’s diversity index (SHDI) value over the years. The observed trend in Jiawang’s eastern region reveals a decline in the quantity of grids exhibiting low SHDI values (below 0.3) that are spatially concentrated in clusters. This decline suggests a rise in the overall diversity of the local landscape. Since 2010, a noteworthy development has been observed in the distribution of high-value grids in addition to those already present in the central and northern mountainous regions and along river peripheries. This emergence of a new cluster of high-value grids has arisen in the northwest. A novel SHDI high-value center has emerged by the year 2020, which is intricately linked to the restoration efforts of coal mining subsidence wetland landscapes.

**Fig. 8:**
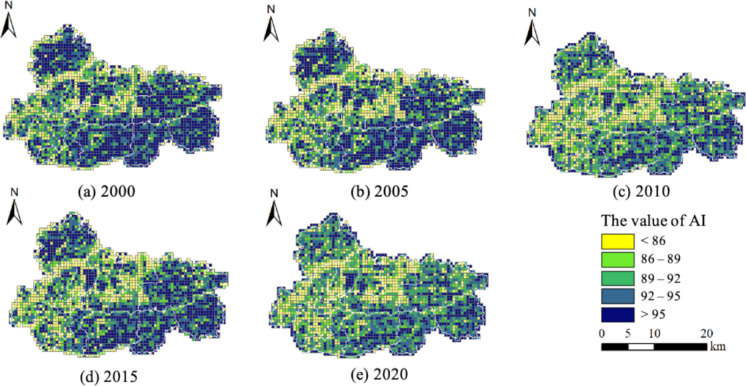
Changes in aggregation index (AI) pattern

The aggregation index (AI) value of Jiawang exhibited dynamic fluctuations during the period from 2000 to 2020, as shown in Fig. 8. Between the years 2000 and 2005, the AI values displayed a notable concentration in the eastern and northwestern regions, while presenting lower values in the peripheral areas surrounding the center urban part. In 2010, there was an increase in the area of regions with low AI values, namely those below 86, in the central and western areas. Conversely, there was a drop in the number of grids exhibiting high AI values, above 95, in the eastern region. Between the years 2010 and 2020, there was a noticeable increase in the geographical extent of low AI value regions in the western areas. This expansion could potentially be attributed to the reclamation of land affected by coal subsidence. However, there was an observed rise in the quantity of grids with high AI values in the eastern and northwestern regions during the year 2015, followed by a subsequent decline by the year 2020. The phenomenon in question can be attributed to the dynamic changes in the landscape of cropland.

### Spatial and temporal changes of habitat quality and spatial autocorrelation analysis

#### Spatial characteristics of habitat quality

**Fig. 9:**
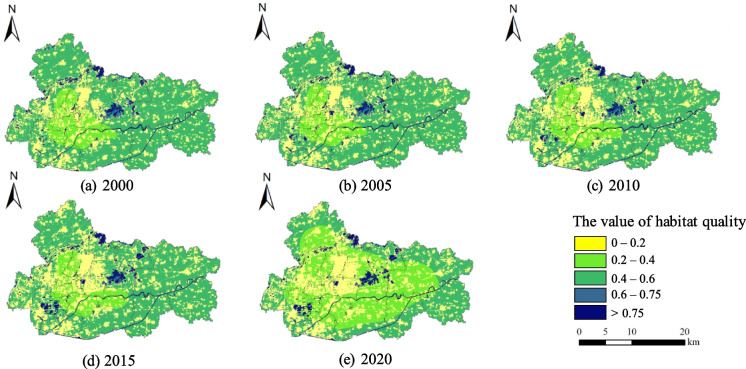
Changes in habitat quality pattern

Figure 9 shows the spatial pattern of Jiawang’s habitat quality from 2000 to 2020. Over the course of two decades, there has been a notable rise in areas characterized by low habitat quality values (below 0.2). This increase has been particularly prominent in the central regions. The emergence of a novel cluster of low-value regions in the southwestern part of Jiawang can be attributed to the development of the central urban area and the establishment of sub-centers. The spatial distribution of sub-low value zones, characterized by values ranging from 0.2 to 0.4, has experienced notable development in Jiawang. Initially concentrated around the dedicated coal mine railways in 2000, these areas have gradually extended to encompass a majority of the region by 2020, with the most significant expansion occurred between 2015 and 2020. This suggests that the growth of urban areas following the exhaustion of resources has a notable influence on the extension of sub-low value areas in terms of habitat quality. Notably, the southwest of Jiawang has gradually formed a new cluster of high habitat quality (greater than 0.75), closely related to the spatial development of the city’s sub-centers in the southwest. This improvement reflects the study area’s efforts as a coal resource-exhausted city to actively pursue and practice urban transformation and development.

#### Transfer analysis of habitat quality

Over the past 20 years, there has been a gradual drop in the average habitat quality of Jiawang, with a decrease from 0.323 in the year 2000 to 0.294 in the year 2020. However, the maximum value of the grid representing the quality of the environment remained constant at 0.949. This suggests that the ecological land cover has not experienced substantial impacts. Table 3 presents the transition matrix illustrating the changes in Jiawang’s habitat quality from the year 2000 to 2020. There was a notable increase in the extent of the habitat quality grid area with values below 0.2, which exhibited a rise from 149.01 km^2^ in 2000 to 192.04 km^2^ in 2020. By contrast, the extent of the habitat quality grid area exhibiting values over 0.75 experienced a small increase, expanding from 22.81 km^2^ in 2000 to 27.91 km^2^ in 2020. It is worth noting that a total area of 149.409 km^2^ consisting of grids with a moderate level of degradation (values ranging from 0.4 to 0.6) had a decline to a lower level of degradation (ranging from 0.2 to 0.4). The majority of these grids that have been degraded are situated in close proximity to the center metropolitan area, thus serving as the primary factor contributing to the overall decrease in habitat quality in Jiawang.

**Table 3:** Habitat quality transfer matrix, unit:km^2^

2000	2020					
	< 0.2	0.2–0.4	0.4–0.6	0.6–0.75	> 0.75	Total
< 0.2	146.95	0.26	0.09	0	1.70	149.01
0.2–0.4	10.12	43.38	0	0.01	1.44	54.95
0.4–0.6	32.93	149.41	189.56	0.02	4.59	376.50
0.6–0.75	1.11	2.57	2.29	0.86	2.02	8.85
> 0.75	0.92	2.08	1.64	0.01	18.16	22.81
Total	192.04	197.71	193.58	0.89	27.92	612.14

#### Results of spatial autocorrelation analysis of habitat quality

As shown in Table 4, the habitat quality of Jiawang demonstrates a significant positive spatial correlation. This is evident from the Global Moran’s indices, all of which exceed 0.4, the *z*-scores, which are all above 40, and the *p*-values, which are all at 0.001. The Global Moran’s I values exhibit an increasing trend over time, suggesting a growing spatial clustering of areas with similar habitat quality. Specifically, this trend indicates that high-quality habitats are increasingly concentrated in specific regions, while low-quality habitats are also clustering together.

**Table 4:** Global moran’s I and *p*-score and *z*-score

Year	Moran’s I	*z*-score	*p*-score
2000	0.43	42.5792	0.001
2005	0.415	41.1453	0.001
2010	0.437	44.0267	0.001
2015	0.471	47.4095	0.001
2020	0.489	50.2603	0.001

**Fig. 10:**
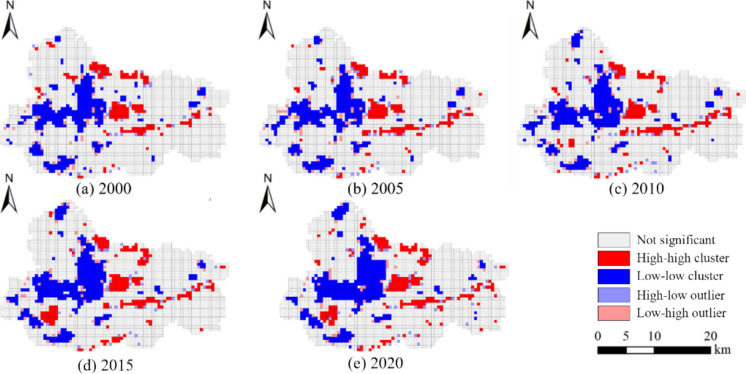
Local indicators of spatial association of habitat quality

The Local Moran’s index analysis provides further insights into the spatial distribution of habitat quality in Jiawang (Fig. 10). High-high clusters typically consist of rivers and mountains, whereas low-low clusters are predominantly composed of center urban areas, townships, and state-owned large coal mining fields. Meanwhile, the range of high-high clusters as well as low-low clusters is consistently broadening. The low-low clusters are exhibiting a phenomenon of diffusion and expansion in proximity to their initial concentrated regions, whereas the high-high clusters have undergone a spatial reorganization, establishing a novel central location in the southwestern region of Jiawang. These findings highlight the substantial spatial variability of Jiawang’s habitat quality and its dynamic evolution over time, underscoring the pronounced spatial heterogeneity and temporal dynamics of habitat quality across the study area.

### Analysis of the impact of landscape metrics on habitat quality

#### Comparison of model performance between GTWR and MGTWR models

The previous analysis has revealed that the habitat quality and landscape patterns of the study area demonstrate spatial and temporal non-stationarity. Thus, GTWR and MGTWR model are employed in this study to investigate the spatiotemporal dynamic impacts of landscape patterns on habitat quality. As shown in Table 5, the MGTWR model, which considers the scale differences of spatiotemporal variables, outperforms the GTWR model and more accurately fits the dynamic response of Jiawang’s habitat quality to landscape patterns. Firstly, the explanatory power (*R*^2^) of the MGTWR model is higher than that of the GTWR model. The *R*^2^ of the MGTWR model is 0.971, an increase of 0.016 compared to the GTWR model. Secondly, the Residual Sum of Squares (RSS) and Akaike Information Criterion corrected (AICc) of the MGTWR model are both superior to those of the GTWR model. The AICc drops from − 42,641.023 in GTWR to − 49,347.072 in MGTWR model, suggesting more key information is retained. Additionally, the lower RSS value of MGTWR model indicates that fewer parameters can achieve regression results closer to the actual values.

**Table 5:** Comparison of diagnostic information between GTWR and MGTWR models

Model	Variable		
	*R*^2^	AICc	RSS
GTWR	0.955	− 42,641.023	47,228.807
MGTWR	0.971	− 49,347.072	30,701.834

#### Scale effects in the spatial and temporal dynamics of landscape patterns on habitat quality

Analyzing the effect of scale on regression outcomes, the results for the GTWR and MGTWR models are shown in Table 6. It is evident that the MGTWR model can identify the optimal spatiotemporal bandwidth for each explanatory variable, whereas the GTWR model has a fixed bandwidth, only reflecting the average spatiotemporal effect of each variable. Specifically, for the MGTWR model, all spatial bandwidths range between 478.5 m and 752.9 m, with a temporal bandwidth of 14.4 years; for the GTWR model, both spatial and temporal bandwidths are 212.3 m and 0.8 years respectively. Therefore, the MGTWR model can effectively measure and identify the scale of spatiotemporal relationships, these varying scales represent the spatiotemporal heterogeneity of the impact of landscape patterns on habitat quality in the study area.

**Table 6:** Comparison of results from GTWR and MGTWR models

Explanatory variable	GTWR						MGTWR				
	bws	bt	βmin	βmed	βmax		bws	bt	βmin	βmed	βmax
NP	212.3	0.8	− 4.999	0.041	7.648		752.9	14.4	− 2.025	0.043	3.288
CONTIG_MN	212.3	0.8	− 5.955	− 0.037	6.880		639.4	14.4	− 4.572	− 0.136	4.846
SHDI	212.3	0.8	− 5.074	− 0.014	6.290		478.5	14.4	− 3.488	− 0.027	4.122
AI	212.3	0.8	− 5.313	− 0.005	7.103		586.6	14.4	− 3.187	0.057	3.277

In terms of spatial scale, the MGTWR model calculated that there are differences in the spatial bandwidths affecting habitat quality for each landscape metric. Lower bandwidth values indicate relatively local changes in the relationships between variables, while higher bandwidth values represent changes across broader spatial extents. The spatial bandwidths of these landscape metrics are all much smaller than the global spatial scale of the study area (the area of the study area is 612 km^2^). This indicates a clear local spatial heterogeneity in the impact of landscape metrics on habitat quality in the study area. Specifically, the spatial bandwidth for NP is the largest at 752.9 m, while for SHDI it is the smallest at 478.5 m, suggesting that SHDI affects habitat quality in the study area at a smaller spatial scale.

In terms of temporal scale, MGTWR model calculated that the temporal bandwidth for each landscape index is 14.4 years. This indicates that the temporal scale of the impact of each landscape metric on habitat quality is the same. Simultaneously, the 14.4-year temporal bandwidth is similar to the time range selected for this study (The study covers the period 2000–2020). This suggests that the impact of these landscape metrics on habitat quality is almost global in terms of time. Furthermore, considering the changes in the standardized regression coefficients, the GTWR model, which uses an average bandwidth estimate, will amplify the range of regression coefficient changes due to the strong local spatial heterogeneity of landscape metrics; for example, the range of SHDI’s standardized regression coefficients in the MGTWR model is − 3.488 to 4.122, while in the GTWR model it is − 5.074 to 6.290. Therefore, considering the scale differences of each variable can lead to more reliable estimation results.

bws is spatial bandwidth in meters; bt is the temporal bandwidth in years; βmin the minimum value of the standardized regression coefficient; βmed is the median value of the standardized regression coefficient; βmax the maximum value of the standardized regression coefficient.

#### Temporal and spatial characteristics of landscape metrics regression coefficients based on MGTWR Model

According to the aforementioned analysis, it is known that within the study period selected for this research, the four landscape metrics have a smaller spatial bandwidth and a larger temporal bandwidth on habitat quality impact. This indicates that such impacts are small spatial extents and display significant local heterogeneity in spatial terms; in temporal terms, they present an almost global scale, with the effects being relatively stable over short periods. Therefore, by analyzing from the temporal and spatial dimensions separately, the specific impact of each landscape metric on habitat quality can be more clearly defined.

**Fig. 11:**
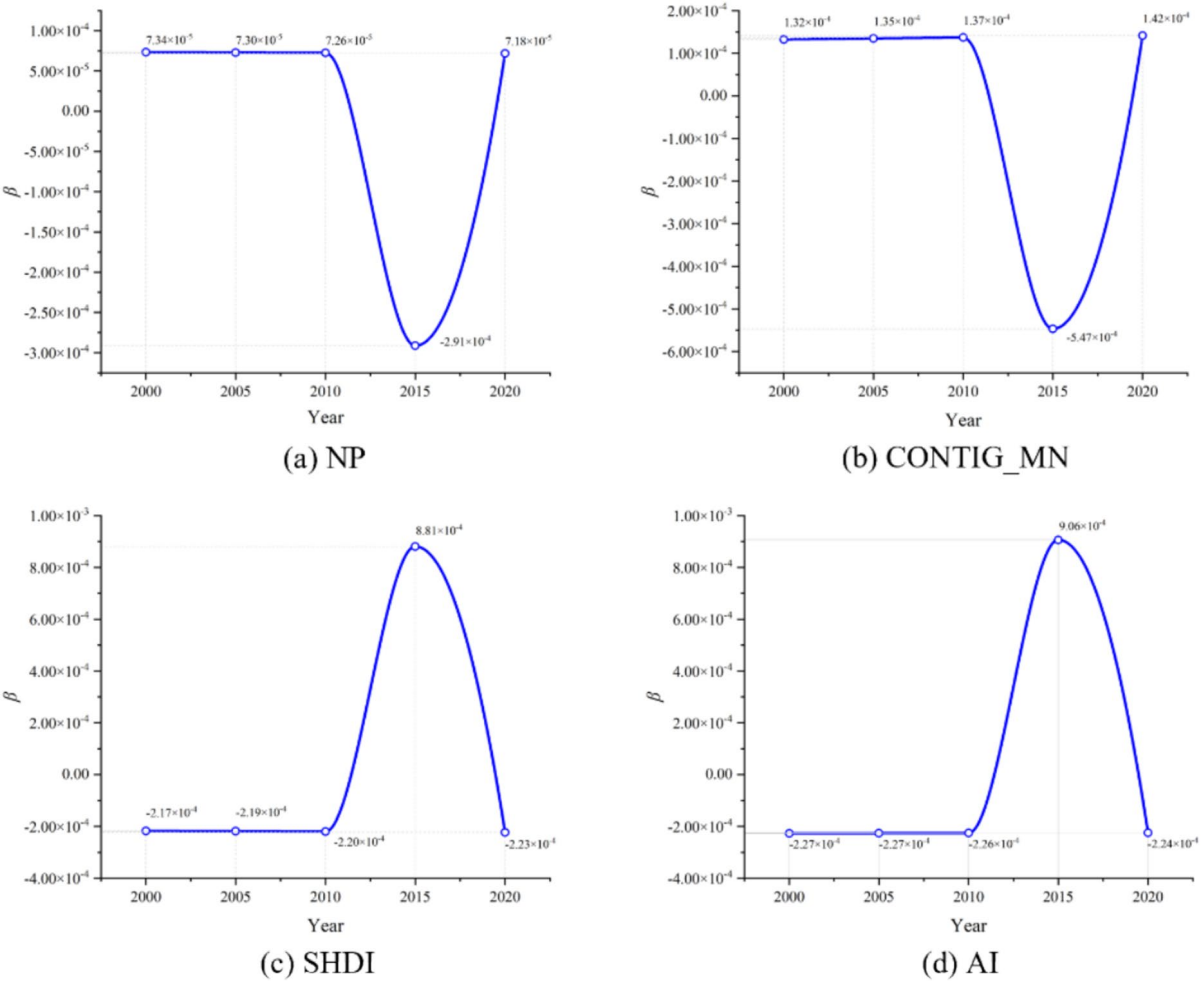
Temporal distribution characteristics of annual average standardized regression coefficients of landscape metrics

Analyzing the changes in regression coefficients over time, the annual average values of the standardized regression coefficients for the four landscape metrics are shown in Fig. 11. It can be seen that there was not much overall change from 2000 to 2020, but there was a significant change in 2015. Specifically, in 2000, 2005, and 2010, the annual average values of the regression coefficients for the four indices tended to be stable. Among them, the annual average values of the standardized regression coefficients for NP and CONTIG_MN were both greater than 0, indicating a positive correlation with habitat quality in the study area; that is, the more landscape patches and the better average connectivity, the higher the habitat quality. In contrast, during this period, the annual average values of the standardized regression coefficients for SHDI and AI were both less than 0, indicating a negative correlation with habitat quality in the study area. That is, during this time, the richer the diversity and the higher the integrity of the landscape in the study area, the lower the quality of habitat manifested. It is noteworthy that in 2015, there were significant fluctuations in the annual average values of the regression coefficients for the four metrics, and the temporal heterogeneity was consistent with the calculated scales. During this period, the annual average values of the standardized regression coefficients for NP and CONTIG_MN became less than 0, while those for SHDI and AI became greater than 0. The possible reason is that 2010–2015 was a period of rapid transitional development in the study area, with the complete closure of coal mines, rapid expansion of impervious surfaces, and the implementation of a series of ecological restoration projects, which are possible reasons for temporal heterogeneity. By 2020, the annual average values of the regression coefficients for the four indices had returned to the levels of 2000–2010, and the transitional development of the study area had become stable.

**Fig. 12:**
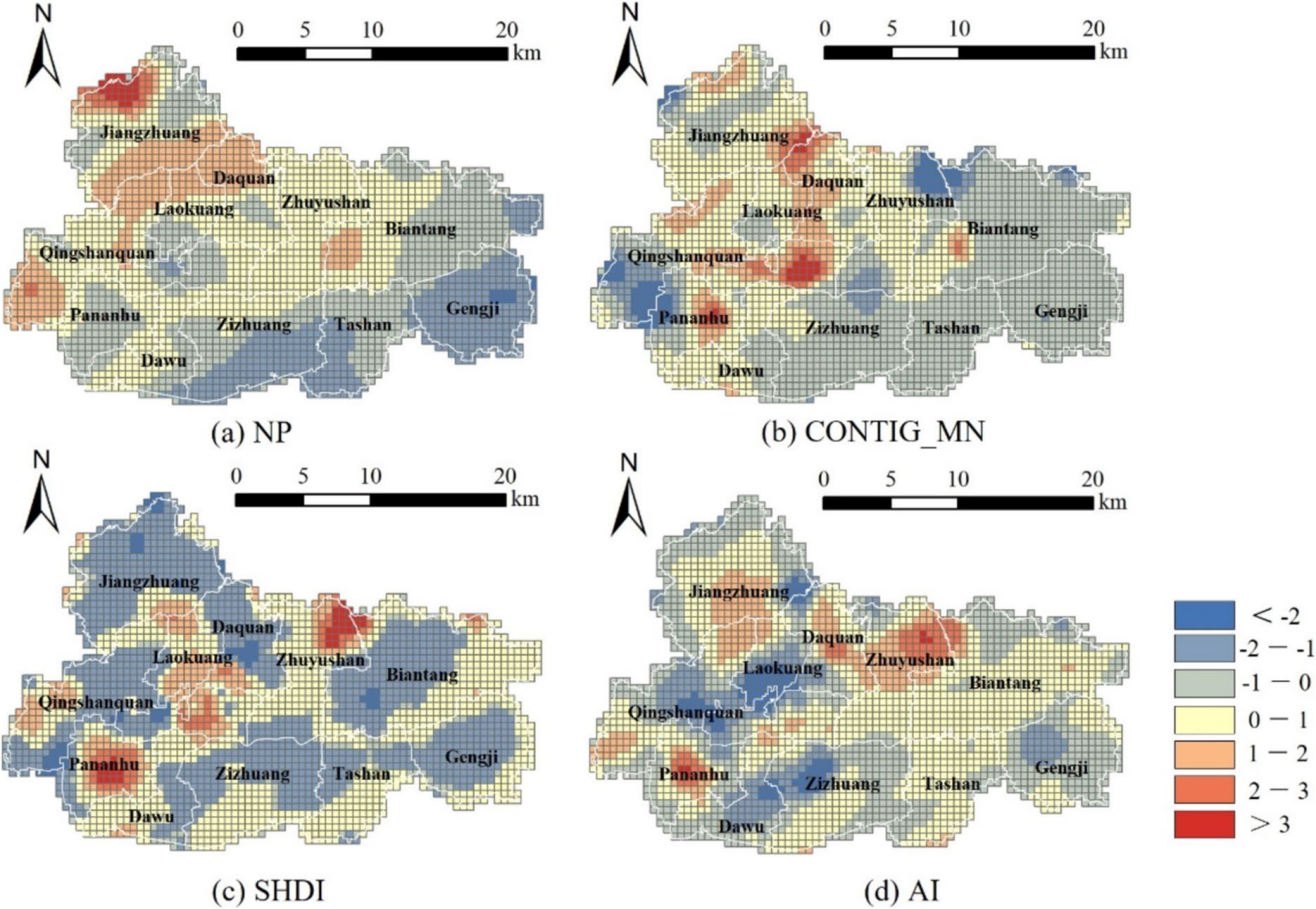
Spatial distribution characteristics of average standardized regression coefficients of landscape metrics

Analyzing the changes in regression coefficients in the spatial dimension, the annual average values of the standardized regression coefficients for the four landscape pattern indices are as shown in Fig. 12. Figure 12a displays the spatial pattern of the average regression coefficients for the NP. It can be observed that NP has a general spatial characteristic of being positively correlated with habitat quality in the northwest and negatively correlated in the southeast. The values of NP in the western and northern parts of Jiawang have the most significant positive correlation with habitat quality, areas which are primarily mountainous and hilly terrain with a large proportion of woodland and grassland. This indicates that the greater the number of woodland landscape patches, the more beneficial it is for the enhancement of habitat quality. In the southeastern region of Jiawang, the NP values have a significant negative correlation with habitat quality, which are mainly large areas of farmland and scattered villages. This shows that the more the number of farmland and rural landscape patches, the more detrimental it is to the improvement of habitat quality. Notably, a small-scale negative correlation between NP values and habitat quality has emerged in the southern edge area of Jiawang’s central urban area and the Pan’an Lake Street, possibly due to new industrial zone construction in the southern central urban area and ecological restoration and sub-center urban development on Pananhu Street after resource depletion in Jiawang. These areas are still under development and construction, and the greater the number of scattered construction land landscape patches, the more disadvantageous it is for the enhancement of habitat quality.

Figure 12b illustrates the spatial relationship between the values of CONTIG_MN and habitat quality, showing an overall pattern of positive correlation in the central part and negative correlation at the edges. The positively correlated grids are mainly distributed in ecological lands such as mountains and waters, indicating that enhancing the connectivity of ecological lands can significantly improve the habitat quality of the study area. Particularly, as a resource-exhausted city, the CONTIG_MN values of landscapes around the central urban area and large coal mines have a significant positive correlation with habitat quality. These areas are disturbed by coal mining and human activities, resulting in a large number of fragmented farmlands and water bodies. If the connectivity of the landscapes in these areas is enhanced, it would significantly improve habitat quality. Conversely, a contiguous negative correlation between CONTIG_MN values and habitat quality is evident in the eastern part of the study area, mainly agricultural production zones, implying that increased connectivity of large areas classified as farmland landscapes does not favor the improvement of habitat quality. Furthermore, we can see that there is a significant negative correlation between CONTIG_MN values and habitat quality in the townscapes far from the central urban area. This indicates that increasing the connectivity of abandoned coal mine industrial plazas and impervious surfaces far from the city center can have a negative impact on habitat quality.

Figure 12c shows the spatial pattern of the average regression coefficients for SHDI. The urban fringe area in the southern part of the central urban area of the study area has a clear cluster of high and positive regression coefficient values, indicating that an increase in SHDI in the central and fringe urban areas contributes to habitat quality improvement. In addition, the SHDI in the central-northern and southwestern parts of the study area has a significant positive correlation with habitat quality. Specifically, before 2010, the southwestern part of the study area consisted of large and fragmented coal mining subsidence wetlands, which, after landscape ecological restoration, have become a national wetland park (Chen et al., 2024); the central-northern part has undergone ecological restoration of quarried depressions. This demonstrates that ecological restoration projects have enhanced landscape diversity and have further had a positive effect on the improvement of habitat quality. The SHDI in the eastern, western, and northern parts of the study area shows a negative correlation with habitat quality, suggesting that higher SHDI values in rural town areas have a negative effect on the improvement of habitat quality.

As shown in Fig. 12d, the negative correlation between AI values and habitat quality in the study area is mainly concentrated in the central and southern parts, where there are large areas of impervious surfaces. This indicates that the higher the level of integration of urban construction land, the stronger the negative impact on habitat quality. Conversely, the agglomeration of ecological lands such as woodlands, grasslands, and water bodies is beneficial for the enhancement of regional habitat quality. The positive correlation between the AI values of the landscape and habitat quality is primarily distributed in the mountainous regions in the northern part of the study area and the wetland landscapes in the south.

## Discussion

### Transformative urban ecological development and changes in habitat quality

Resource-exhausted cities have played a crucial role in China’s economic development, with coal-related resource-exhausted cities accounting for more than half. These cities have increasingly faced numerous contradictions and problems during their development due to reasons such as the gradual depletion of resources (Dowling et al., 2018; Yu et al., 2019). Among these issues, the destruction of the ecological environment caused by coal mining and human activities has received the widest attention (Zhang & Yu, 2023). Under the guidance of the national ecological civilization strategy, the ecological restoration and transformational development of cities are imperative.

During the ecological transition period, the implementation of ecological restoration projects can effectively mitigate the damage to land and the ecological environment caused by mining activities (Tong et al., 2017; Wortley et al., 2013). Our research has found that fragmented and isolated coal mining subsidence wetlands have been connected and integrated into larger lake wetlands, improving habitat quality and creating new regional ecological habitats. This is consistent with the findings of many scholars’ previous research (Grayson et al., 1999; Klaus & Kiehl, 2021). However, this study found that the implementation of spot ecological restoration projects can only improve local habitat quality. Due to the substantial financial investment and time cost required for ecological restoration projects, the speed of urban construction land expansion far exceeds the implementation speed of ecological restoration projects in the context of rapid urbanization. The construction land in the central urban areas of the city continues to expand, with an increasing population. Furthermore, driven by land finance and urban planning, the construction of new sub-centers adjacent to ecological habitats further affects the regional habitat quality. Ultimately, the average value of the overall habitat quality of the city is on a downward trend. Therefore, the ecological transformation of coal resource-exhausted cities needs to consider the relationship between ecological restoration, protection, and urban construction from a holistic regional perspective, so as to enhance the overall habitat quality and improve the socio-ecological system structure.

### Advantages of the study methodology

The impact of landscape patterns on habitat quality has been widely concerned. However, research related to coal resource-exhausted cities, which urgently require ecological environment improvement, is still lacking. The biggest difference between these cities and ordinary cities is their strong reliance on the development of coal resources for urban development, and they suffer from more serious ecological environment destruction (S. Li et al., 2021a, 2021b). Therefore, when calculating habitat quality using the InVEST model, in addition to conventional land cover type threat factors, we specifically selected state-owned large coal mine industrial squares, coal mining dedicated railways, and other railways as threat factors to more accurately reflect the characteristics of habitat quality in coal resource-exhausted cities. Furthermore, the response of habitat quality to landscape patterns is a long-term process, where the consideration of spatiotemporal heterogeneity is necessary. At the same time, scale plays a critical role in understanding and explaining ecological processes and patterns. This includes not only the spatial scale but also the temporal scale. Therefore, this study innovatively used the MGTWR model to explore the spatiotemporal dynamic impact of landscape patterns on habitat quality and its scale effects. This helps to clarify the complex relationship between the landscape patterns and habitat quality of coal resource-exhausted cities.

### Landscape pattern planning strategies from the perspective of habitat quality enhancement

Although urban planners have noticed and tried to enhance the habitat quality of coal resource-exhausted cities by implementing ecological restoration projects or optimizing high-quality habitat patches, few have considered how to improve the region’s habitat quality from the perspective of the overall landscape pattern. Based on the research results of habitat quality’s dynamic response to landscape patterns and scale effects, this study proposes the following urban landscape planning strategies:

From a perspective of the entire national territory space, plan the overall pattern of national land space protection and restoration. Focus on the important natural landscapes within the region, build an ecological security pattern, and enhance the overall ecological resilience. Furthermore, on the basis of the ecological security pattern, delineate protected restoration zones. Our research results indicate that there is significant local spatial heterogeneity in the impact of landscape indices on habitat quality. Therefore, targeted planning strategies need to be developed for different zones.

For urban central areas dominated by construction land, reduce the landscape aggregation of multiple high-density urban centers by breaking down the construction land to make it as fragmented as possible. This will increase the number of patches, thereby reducing their positive impact on habitat quality, while embedding high-quality habitat patches such as woodlands and enhancing their connectivity, which will also increase landscape diversity and strengthen their positive impact on the habitat. As the urbanization process accelerates, the positive effects of reducing urban construction land aggregation and increasing the connectivity of high-quality urban habitat patches will become increasingly significant.

For the peripheral areas of the central urban district and the sub-central areas, construction land patches constantly transform the original farmland, making the landscape pattern more complex. Increasing landscape diversity and connectivity can buffer against habitat degradation. Reducing landscape aggregation, while lowering the density of urban sprawl and reasonably preserving high-quality habitat patches, will reduce the encroachment of construction land on other patches and improve habitat quality to some extent. This will also have a positive impact on urban habitat quality.

For small towns and rural farmland areas, the landscape pattern is relatively stable. High-intensity, large-scale changes in the landscape pattern can cause habitat quality decline in these areas. We should moderately reduce the degree of landscape fragmentation and increase integration while maintaining the status quo. This can positively affect habitat quality to some extent. Furthermore, enhancing the landscape diversity of rural farmland areas and reducing the landscape diversity of town areas can improve habitat quality to some extent.

For coal resource-based cities, the landscape pattern of mining sites deserves special attention. Special care should be taken to mitigate the negative impact of landscape fragmentation on habitat quality during ecological restoration and landscape ecological reconstruction in these places. The enhancement of landscape diversity, connectivity, and integration all have positive roles in improving habitat quality.

Moreover, according to our research results: the temporal scale of the landscape pattern’s impact on habitat quality is 14.4 years. Therefore, the cycle of landscape pattern planning strategies based on habitat quality improvement can be set to 15 years. This is consistent with the cycles of higher-level planning such as national spatial planning, which is more conducive to the implementation of planning strategies.

### Contributions and limitations

Our study specifically focused on the ecological transition of coal resource-exhausted cities, innovatively exploring the scale effects of landscape patterns on the spatiotemporal dynamic impacts on habitat quality. However, our study has limitations. Firstly, the calculation of landscape metrics was based on spatial units with a resolution of 500 m × 500 m, and it needs to be clarified whether the appropriate grid scale is applicable to other coal resource-exhausted cities, such as the modifiable areal unit problem (Li et al., 2023a, 2023b). Secondly, we chose land cover data every five years as the temporal structure, which may miss capturing minor changes within these five years, thus affecting the temporal scale (Hu et al., 2022). For further research, we recommend considering shorter time intervals to reveal the temporal dynamics of landscape patterns and habitat quality. Moreover, the calculations of landscape patterns and habitat quality were based on land cover data with 30-m resolution, and higher resolution data could be used in the future. Lastly, in exploring the relationship between landscape patterns and habitat quality, we mainly used linear regression models. However, some studies suggest that this relationship might be non-linear. Future research should explore this non-linear relationship, possibly using non-linear regression models or machine learning methods (Li et al., 2023a, 2023b; Mitchell et al., 2015), to provide a more comprehensive understanding for ecosystem management and the sustainable transformation of resource-based cities.

## Conclusion

This study presents a comprehensive methodology for comprehending the dynamic influence of landscape patterns on habitat quality, especially in cities undergoing transitions due to resource depletion. Focusing on Jiawang, a typical coal-depleted city in eastern China, we revealed the multifaceted impacts of landscape patterns on habitat quality across various spatiotemporal scales. The results of this study have enhanced our comprehension of the intricate relationship between the evolving urban landscape patterns and habitat quality after severe resource depletion. The main conclusions drawn from the research are as follows:

Firstly, after coal resource exhaustion, the landscape patterns in Jiawang underwent a notable process of urbanization, characterized by a rapid expansion of impervious surfaces. The landscape metrics, namely NP, CONTIG_MN, SHDI, and AI, exhibited clear spatiotemporal non-stationarity throughout the 20-year period.

Secondly, over the course of these two decades, there were notable temporal fluctuations in habitat quality. The expansion of impervious surfaces in the city led to an overall decline in habitat quality. However, the implementation of ecological restoration projects has given rise to distinct clusters of high-value habitat quality in Jiawang.

Thirdly, the MGTWR model, which places emphasis on nuanced variations in spatiotemporal heterogeneity, exhibited superior performance compared to the GTWR model. The study emphasized the spatial and temporal variations in the influence of each landscape metric on habitat quality, offering a more extensive and intricate depiction of the intricate and evolving connection between the two factors. The regression results of Jiawang’s landscape patterns in relation to habitat quality revealed clear spatial heterogeneity at a small spatial extent. However, over a short period of time, the temporal heterogeneity remained relatively stable.

Moreover, it is recommended that, from the perspective of the entire national territorial space, different zones be delineated based on the local spatial heterogeneity of the impact of various landscape indices on habitat quality, and targeted strategies be formulated accordingly; based on the global temporal scale of the impact of landscape patterns on habitat quality, landscape pattern optimization strategies should be linked with higher-level national spatial planning; grounded in the uniqueness of coal resource-exhausted cities, optimize the landscape patterns of mining sites.

In summary, these research findings offer crucial theoretical support and practical guidance for sustainable spatial planning strategies. They emphasize the importance of integrating ecological restoration with urban development, particularly in regions experiencing resource depletion. Such insights contribute to building a harmonious coexistence between urban and ecological environments, ensuring a balanced approach to development and conservation.

## Data Availability

Data will be made available on request.
