# Correction to: Impacts of landscape patterns on habitat quality in coal resource‑exhausted cities: Spatial–temporal dynamics and non‑stationary scale effects

**DOI:** 10.1007/s10661-025-14264-3

**Published:** 2025-06-26

**Authors:** Zixuan Li, Ziqi Xu, Yedong Chen, Sihao Gu, Cheng Li

**Affiliations:** 1 Leibniz Institute of Ecological Urban and Regional Development, 01217 Dresden, Germany; 2 School of Architecture and Design, China University of Mining and Technology, Xuzhou, 221116 China; 3 School of Architecture, Southeast University, Nanjing, 210096 China; 4 School of Mechanics and Civil Engineering, China University of Mining and Technology, Xuzhou, 221116 China; 5 School of Architecture and Urban Planning, Nanjing University, Nanjing, 210093 China

**Correction to** **:** **Environ Monit Assess (2025) 197:297**

10.1007/s10661-025-13707-1

The article Impacts of landscape patterns on habitat quality in coal resource‑exhausted cities: Spatial–temporal dynamics and non‑stationary scale effects, written by Zixuan Li, Ziqi Xu, Yedong Chen, Sihao Gu and Cheng Li, was originally published under exclusive license to © The Author(s), under exclusive licence to Springer Nature Switzerland AG 2025. As a result of the subsequent decision to publish the article under the open access model, the article’s copyright notice was changed on June 13, 2025 to © The Author(s) 2025 and the article is now distributed under a Creative Commons Attribution 4.0 International License, which permits use, sharing, adaptation, distribution and reproduction in any medium or format, as long as you give appropriate credit to the original author(s) and the source, provide a link to the Creative Commons licence, and indicate if changes were made. The images or other third party material in this article are included in the article’s Creative Commons licence, unless indicated otherwise in a credit line to the material. If material is not included in the article’s Creative Commons licence and your intended use is not permitted by statutory regulation or exceeds the permitted use, you will need to obtain permission directly from the copyright holder. To view a copy of this licence, visit http://creativecommons.org/licenses/by/4.0/.

